# Efficacy of platelet-rich-fibrin for the treatment of alveolar osteitis: a systematic review and meta-analysis

**DOI:** 10.4317/medoral.26801

**Published:** 2025-03-23

**Authors:** Camila Ávila-Oliver, Valentina Veloso, Germán Laissle, Ana María Rojas, Francisca Verdugo-Paiva, José Ramos-Rojas

**Affiliations:** 1 Dental Department, Facultad de Odontología y Ciencias de la Rehabilitación, Universidad San Sebastián, Santiago, Chile; 2 Epistemonikos Foundation, Santiago, Chile; 3 Dental Department, Facultad de Medicina Clínica Alemana Universidad del Desarrollo, Santiago, Chile; 4 Department of Surgery, Clínica Bupa Santiago and Hospital Exequiel Gonzalez Cortes, Santiago, Chile; 5 Orofacial Pain and TMD Program, Facultad de Odontología, Universidad Andrés Bello, Santiago, Chile

## Abstract

**Background:**

This systematic review aims to provide an updated summary of the available evidence on the role of platelet-rich fibrin (PRF) in the treatment of alveolar osteitis.

**Material and Methods:**

Searches were conducted in several electronic databases, including PubMed, EBSCO, Cochrane Central Register of Controlled Trials (CENTRAL) LILACS and ClinicalTrials.gov. No date or language restrictions were applied. Two reviewers independently evaluated eligible studies according to predefined criteria and extracted data using a standardized form. Meta-analyses were performed to estimate results and the certainty of evidence, using the GRADE approach, was assessed.

**Results:**

The search strategy yielded 1.706 references. Finally, 4 randomized trials were included and assessed quantitatively. Overall, the risk of bias was low for 75% of the domains reviewed across studies. The studies included a total of 179 patients, where the intervention group received PRF, and the control group received several treatment alternatives, including iodoform gauze, zinc oxide eugenol, and saline solution. Results showed that the use of PRF may decrease pain severity measured on day 3 (MD -1.66, CI 95%, -4.11 to 0.78) and on day 7 (MD -1.57, CI 95%, -4.00 to 0.88), and improves alveolar socket healing (SMD 2.25; 95% CI 1.70 to 2.80; *p*<0.00001; I2=12%).

**Conclusions:**

The results of this study demonstrate that PRF improves alveolar healing, reduces analgesic use in patients, and likely increases overall clinical efficacy, making it a valuable alternative in the treatment of alveolar osteitis. Despite these findings, this review also showed a great degree of uncertainty on the impact of PRF on pain severity associated with alveolar osteitis. Although these results are promising, further randomized clinical trials with standardized methodologies must be performed to validate these findings.

** Key words:**Systematic review, platelet-rich fibrin, dry socket.

## Introduction

Alveolar osteitis, more commonly known as ‘dry socket,’ is a prevalent oral complication that can often occur after lower third molar extraction (1) and corresponds to the inflammation of the alveolar socket due to failure of the alveolar healing mechanisms (2). Alveolar osteitis is characterized by intense and localized postoperative pain and may or may not be accompanied by halitosis and the presence of a partially or completely disintegrated clot within the alveolus (3). A significant proportion of patients, around 45%, require multiple postoperative visits to manage the condition effectively (4,5). Furthermore, one study found that individuals with dry socket required up to four visits to manage the symptoms (6). This has direct implications for individuals, particularly on the associated cost derived from multiple postoperative care visits to treat the condition.

Treatment alternatives focus on providing symptomatic relief and include two main approaches (7). The first one consists of debris removal from the socket by applying saline solution irrigation and the use of analgesic medication (3,8). There are several intra-socket medications, including antibacterials, topical anesthetics, and obtundents, or a combination of all three(3). The routine intra-alveolar medication generally consists of alvogyl paste, whose primary components are eugenol, butamben, and iodoform (8,9); zinc oxide eugenol (ZOE), which has sedative properties and iodoform gauze impregnated with 5% eugenol (5). The second alternative includes the use of autologous platelet concentrates (7), such as platelet-rich fibrin (PRF), which has gained attention as a potential therapeutic option since it promotes epithelization of the alveolar socket, improving bone coverage and allowing optimal healing (7,10). Preparation is simple and presents minimal risk to the patient (11,12). The application of PRF delivers a concentrated source of healing factors directly to the extraction site, enhancing outcomes beyond mere symptom relief. It promotes faster tissue regeneration, reduces pain intensity and duration, and decreases the risk of secondary complications (5,13-15).

In dentistry, extensive research has been conducted on PRF, as a highly success alternative for preventing alveolar osteitis, following third molar extraction (16-18). However, its application as a treatment option is still being discussed. A recent systematic review examined the efficacy of PRF in managing alveolar osteitis (AO), finding consistent evidence for PRF’s effectiveness in reducing pain and promoting wound healing (19). However, most of the included studies evaluated PRF as a preventive measure to reduce the incidence of AO (14,20-22), and no quantitative analysis was performed.

Results from different studies have demonstrated a reduction in pain and an accelerated healing process of the alveolar socket (15,23-25). However, other studies reported no significant advantage in pain reduction (26,27). These contradictory results have implications for the use of PRF in clinical practice.

Although the use of FRP as a treatment alternative is relatively new, it is important to highlight that since its appearance, it has continuously demonstrated a regenerative effect that has allowed it to be used not only in dentistry but also in general medicine (28).

The present study aims to conduct a systematic review of the available evidence to evaluate the efficacy of PRF in the treatment of alveolar osteitis, a frequent complication of dental extractions that is characterized by severe pain. Therefore, its effective management could potentially reduce postoperative morbidity. In this context, a systematic review of the best high-quality evidence could provide valuable insights and guidance for clinical decision-making processes regarding the implementation of PRF as a therapeutic modality for alveolar osteitis in dental practice.

## Material and Methods

- Study protocol and registration

This manuscript complies with the ‘Preferred Reporting Items for Systematic Reviews and Meta-Analyses’ (PRISMA) guidelines for reporting systematic reviews and meta-analyses (29). A protocol was established according to the evidence-based PICO model (population, intervention, comparison, and outcome) to answer the following question: “What is the efficacy of platelet-rich fibrin (FRP) in the treatment of dental alveolitis?”. All authors reviewed the protocol and registered in the International Prospective Register of Systematic Reviews (PROSPERO) (registration no. CRD42024528381).

- Eligibility criteria

To conduct the study selection, the following inclusion criteria were established: 1) Study design: the study included only randomized controlled trials (RCTs) reporting outcomes on dental alveolitis. Studies evaluating the effects in animal models or *in vitro* conditions were excluded. 2) Population: Participants had to undergo routine or complex dental extraction of one or more permanent teeth using local or general anesthesia. There were no exclusions due to participants' age. Patients who reported smoking were included. Studies performed in immunocompromised patients with a medical condition or co-morbidity that could influence the healing process of oral tissues were excluded. 3) Intervention: The evaluated intervention was the use of platelet-rich fibrin (PRF) for the treatment of alveolar osteitis. 4) Comparators: The intervention was compared with any other conventional treatment used for alveolar osteitis. 5) Outcomes: The primary outcome of interest was pain assessment and alveolar socket healing. Secondary outcomes were restricted to swelling, analgesic consumption and adverse events.

- Information sources

A comprehensive search was performed on several electronic sources, including Cochrane Central Register of Controlled Trials (CENTRAL), PubMed, EBSCO of dental and oral sciences databases, LILACS, and ClinicalTrials.gov (https://clinicaltrials.gov). The searches covered from database inception to December 2023. No date, filters or language restrictions were applied. The search strategy used in the main databases is presented in Supplement 1 (10.5281/zenodo.13830231). Cross-citation searches using Google Scholar were conducted using the references of the included studies.

- Selection process

Two independent reviewers screened the retrieved articles by their titles and abstracts. The included studies were selected in full text by duplicate against the inclusion criteria. Disagreements between reviewers of the included articles were solved by consensus or, if necessary, by a third author. The final included articles were recorded in RevMan 5.4. The selection process was documented in a PRISMA flow diagram (29). 

- Data collection and extraction 

Data extraction from the included articles was performed using a standardized form, by duplicate and disagreements were solved by consensus before data entry into RevMan version 5.4. The following information was recorded from each reference: general characteristics of the study (country of origin, funding source, study design, inclusion, and exclusion criteria), characteristics of participants in the study, including the number of patients evaluated in each group, surgical site, mean age, and sex. Additionally, the type of medication used in the control group, platelet derivate preparation, and primary and secondary outcomes including types of scales, measures used, and follow-up times) will be recorded.

- Risk of bias assessment 

The quality of the RCTs was evaluated using the Cochrane risk-of-bias tool (RoB 2) (30). The assessment included the following criteria: 1) bias arising from the randomization process; 2) bias due to deviations from the intended interventions; 3) bias due to missing outcome data; 4) bias in the measurement of the outcome; 5) bias in the selection of the reported results. All studies were assessed by duplicate and domains were rated as either high, low, or unclear. After, the risk of bias was tabulated for each included study, along with a judgment of low, high, or unclear RoB for each domain.

- Statistical analysis

To measure the treatment effect in dichotomous outcomes, the estimate of the treatment effect of an intervention was expressed as risk ratios (RR) along with the 95% confidence intervals (CI) (31). For continuous outcomes, the mean difference and standard deviation (SD) were used to summarize data, together with the 95% CI. Whenever continuous outcomes were measured using different scales, the treatment effect was expressed as a standardized mean difference (SMD) with 95% CI (31). Risk ratios for dichotomous data and mean differences for continuous data using the inverse variance method were combined with the random effects model. A narrative summary is presented when combining outcome data, which was not feasible due to differences in the reported outcomes. Statistical heterogeneity was assessed by visually inspecting the forest plots, considering the χ2 test (with the significance level set at *P* < 0.10), and using the I2 statistic. Where statistical heterogeneity was moderate, substantial, or high (I2 > 75%) or where there was clinical heterogeneity, possible causes were investigated by exploring the impact of participant characteristics or other variables. Afterward, these results were displayed in the 'Summary of Findings Table' as a mean difference (32).

- Certainty of the evidence assessment

The authors independently assessed the certainty of the evidence for all outcomes by using the Grading of Recommendations Assessment, Development and Evaluation working group methodology (GRADE Working Group) (31), across the domains of risk of bias, inconsistency, indirectness, imprecision and reporting bias. Certainty was adjudicated as high, moderate, low, or very low for the main outcomes. The results are presented in a Summary of Findings (SoF) Table using the GRADEpro software.

## Results

- Results of the search

**Figure 1:**
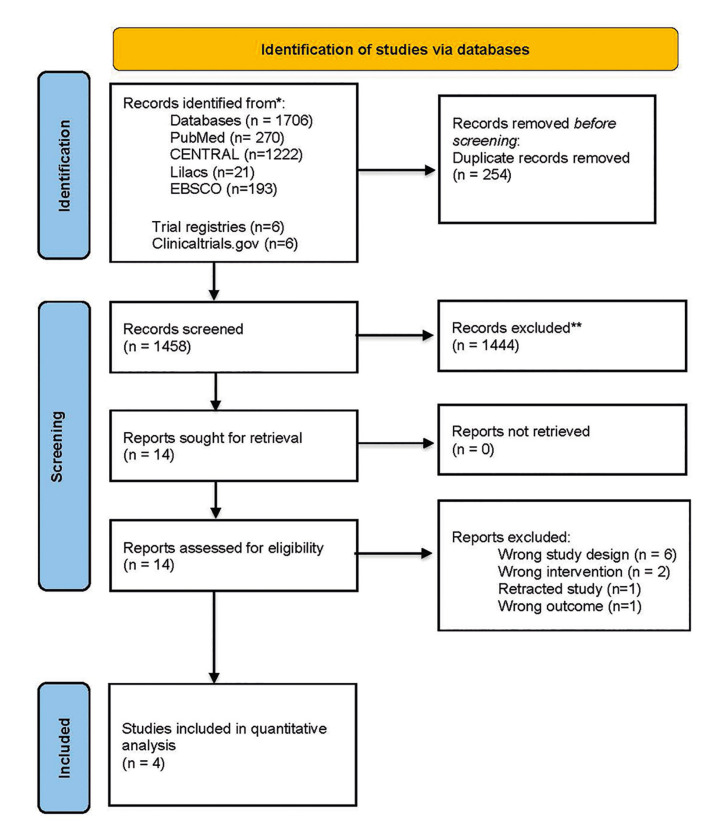
PRISMA Flowchart.

The flowchart (Fig. 1) illustrates the article selection process conducted for this review. A comprehensive search strategy yielded 1,712 records. After removing duplicates, 1,458 articles were available for selection by screening their title and abstracts. Among these, 1,444 articles were excluded against previously defined inclusion criteria. A total of 14 articles were assessed by full text. Finally, 4 trials were included in the review. Additionally, Supplement 2 (https://zenodo.org/records/13830320) provides a list of trials excluded and the reasons for exclusion.

- Description of the included studies

The four included trials had a parallel-group design (8,24,26,32). Additionally, all studies took place in hospital or clinical settings. Two of the selected studies were conducted in India (8,26), and the other two in Turkey (24) and China (32), respectively. Participants were aged from 18 to 60 years, and mostly were women. Three trials specified the clinical criteria used for alveolar osteitis diagnosis, including continuous, severe and radiating pain as the onset of symptoms 1-3 days post extraction (24,26,32). The intervention group received PRF and the control group included alveogyl (8), iodoform gauze (32), zinc oxide eugenol (ZOE) (26) and saline solution (24).

Two studies evaluated the condition in mandibular third molars (24,32), Hussain (26) evaluated alveolar osteitis on mandibular molars and Keshini (8) did not specify. The characteristics of the included studies and the demographic information of the study participants are described in Table 1 and Table 2.

- Risk of bias assessment

**Figure 2:**
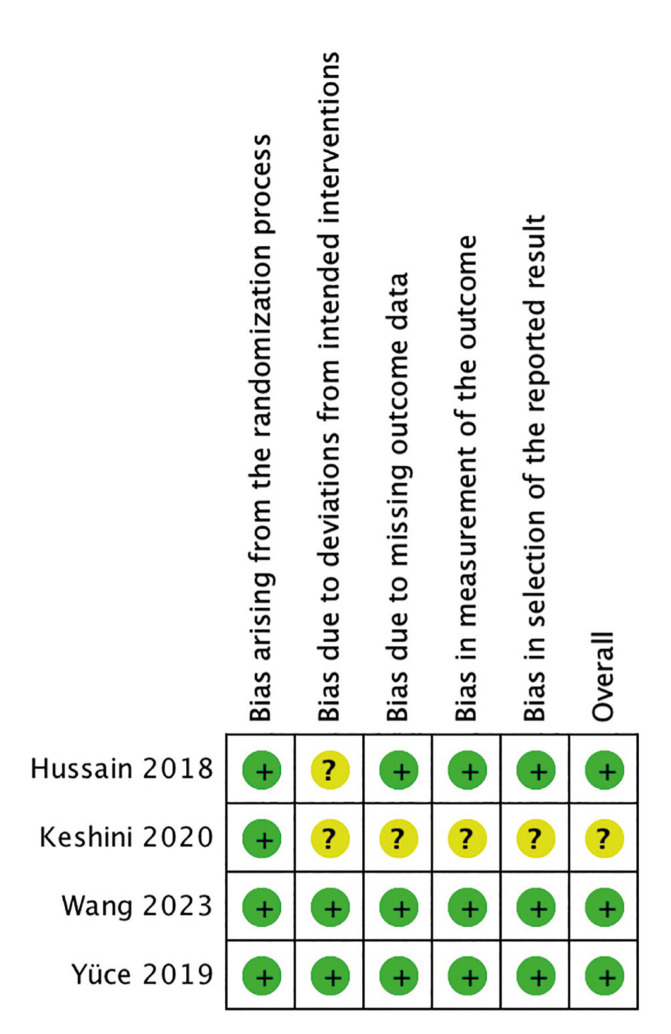
Risk of bias assessment across the included studies in the review.

Three of the four included studies were assessed as having a low risk of bias across the different domains. The remaining trial presented some considerations through the criteria of bias due to deviations from the intended interventions, bias due to missing outcome data, bias in the measurement of the outcome and bias in the selection of the reported results. Therefore, it was rated overall as ‘unclear’. Figures 2, illustrate the risk of bias assessment across the included studies.

- Effects of intervention

Pain severity on days 3 and 7: Four studies, including a total of 179 patients, reported a reduction in pain severity on day 3 and day 7 (8,24,26,32). Use of PRF was associated with a reduction of 1.66 points in the visual analog scale (MD -1.66; 95% CI: -4.11 to 0.78; *p*<0.00001; I2= 98%) when compared to ZOE, saline solution, alvogyl or iodoform gauze measured on day 3. For day 7, the pooled estimate MD was -1.57 (95% IC -4.00 to 0.88; *n*=140; *p*<0.00001; I2=99%). The certainty of the evidence was assessed as very low.

**Figure 3:**
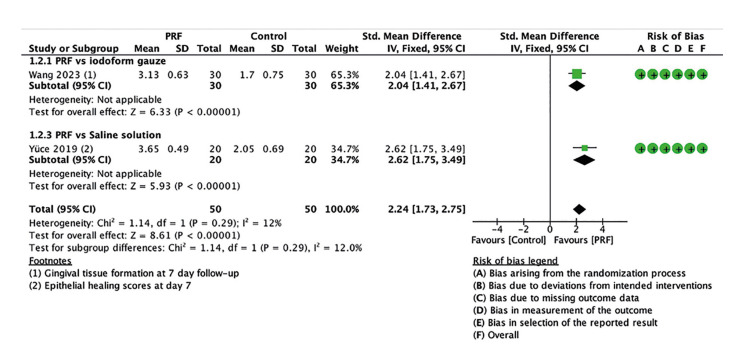
Forest plot showing the effect of PRF vs. control on alveolar osteitis for alveolar socket healing measured at day 7.

Alveolar socket healing: Alveolar socket healing was measured on a rating scale from 0 to 4 in one trial (32) and from 0 to 3 in the other (24); both scales assessed soft tissue formation over the alveolar bone within a follow-up period of seven days. Two trials, including 100 patients, reported improved alveolar socket healing. Fig. 3 shows the use of PRF was associated with an improvement of 2.25 points (SMD 2.25; 95% CI 1.70 to 2.80; *p*<0.00001; I2=12%). The certainty of the evidence was classified as moderate.

**Figure 4:**
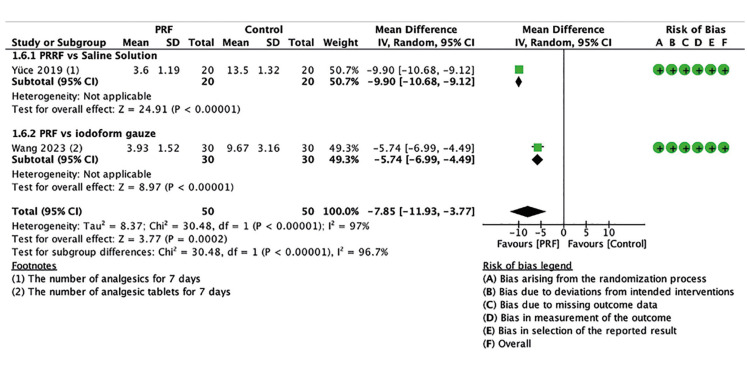
Forest plot showing the effect of PRF vs. control on alveolar osteitis for analgesic use measured at day 7.

- Analgesic use

Analgesic use was measured as the number of Tablets taken within a 1-week period after treatment (24,32). Two trials, including 100 patients, reported reduced analgesic consumption. Fig. 4 shows the use of PRF was associated with a decrease of 7.85 fewer Tablets (MD -7.85; 95% CI -11.93 to -3.77; *p*<0.00001; I2=12%). The certainty of the evidence was assessed as moderate.

- Clinical efficacy

The overall clinical efficacy was measured only in one trial (32), defined as the degree of pain relief and bone wall pressure in the alveolar socket within a follow-up period of 1 week after treatment. Based on this, patients were classified as ‘cured’, indicating pain relieved, with no obvious tenderness in the alveolar bone wall; ‘effective’, indicating pain relieved accompanied by mild tenderness in the alveolar bone wall; and ‘ineffective’, indicating no pain relief, with obvious tenderness in the alveolar bone wall. One trial, including 60 patients, reported an improved clinical efficacy. The use of PRF was associated with 1.56 times higher clinical efficacy compared to control (RR 1.56; 95% CI 1.14 to 2.12; *p*=0.005). The certainty of the evidence was classified as moderate.

To explore heterogeneity, we systematically excluded each study one by one and then combined the results of the remaining studies in each iteration. Although no significant changes were observed in heterogeneity, this could be attributed to clinical differences in the interventions included as comparators. A particular difference can be observed in the outcome results when a saline solution is used in the control group, where pain intensity and analgesic use are notably reduced.

- Summary of Findings Table

A summary of findings with the certainty of the evidence using GRADE considerations (31) is described in Table 3 (32).

## Discussion

The main objective of this review was to assess the effectiveness of PRF for the treatment of alveolar osteitis compared to other available therapeutic options. Our analysis included evidence from four high-quality randomized controlled trials, involving a total of 179 participants, comparing PRF to four different interventions in control groups.

Despite the limited number of participants, the results indicate that PRF may be comparable or even superior to other treatment alternatives in terms of clinical efficacy. Two RCTs provided moderate-certainty evidence suggesting that PRF enhances alveolar socket healing relative to other treatments, alongside a reduction in pain severity. However, the evidence supporting pain relief was of very low certainty, limiting the confidence in these findings. Interestingly, two of the studies documented reduced analgesic use when PRF was administered, a result supported by moderate-certainty evidence.

Our review underscores the need for additional well-designed clinical trials with larger sample sizes and extended follow-up periods to validate PRF as a standard treatment for alveolar osteitis. To date, no other systematic reviews have assessed the effectiveness of PRF specifically for this purpose, highlighting the novelty of our findings and their potential utility for dental practitioners. This review identifies a significant evidence gap concerning therapeutic approaches for established dental alveolitis.

Recently, Laforgia *et al*. (19) conducted a systematic review that focused on the incidence of alveolar osteitis, or “dry socket,” primarily addressing preventive strategies rather than treatment. Their findings advanced the understanding of prevention, yet a significant gap persists regarding the treatment of patients who already developed this painful condition. Our systematic review aims to address this gap by evaluating the efficacy of PRF in treating patients with established dry socket, offering a comprehensive analysis that may lead to more effective treatment protocols.

A panoramic review published in 2023 (33) examined both randomized and non-randomized clinical studies investigating the effect of PRF in managing pain associated with alveolar osteitis. This review reported findings that are consistent with ours, indicating a consistent trend toward pain reduction across all included studies. However, due to its exploratory nature, the review did not conduct assessments of bias risk, meta-analysis, or certainty of evidence evaluations. Similar to our findings, other authors have reported statistical differences in pain severity, demonstrating the benefit of PFR (25).

An interesting finding of our review is that sensitivity analysis revealed significant results when comparing PRF with saline solution (control) (24). Despite the limited number of participants, PRF showed a positive effect in reducing pain and analgesic use. This suggests that PRF may effectively reduce pain compared to interventions lacking analgesic effects, and it demonstrates a promising trend for better pain outcomes compared to other alternatives. Additionally, PRF's impact on clinical improvement and tissue damage resolution appears comparable to other therapeutic options, potentially yielding similar results. Notably, treatment with ZOE appears to offer better outcomes for pain management.

However, this review has limitations, specifically regarding individual outcomes combined in the meta-analysis that should be carefully evaluated. While the results reported suggest an overall benefit in the use of PFR, the high level of inconsistency in the studies limits the reliability of their conclusions. The considerable heterogeneity observed across the studies can be attributed to the variety of treatment alternatives used in the control groups. Currently, there are various therapeutic alternatives for dental alveolitis, each with different mechanisms of action, which makes them heterogeneous and challenging to compare (33). Furthermore, the analysis pooled results were obtained from a limited number of trials with a small number of participants. Future studies should address these limitations with more well-designed RCTs, higher sample sizes, and standardized treatments for control groups.

Considering that pain is the most challenging symptom of dental alveolitis, primary treatment approaches focus on its management. Options include alveolar lavage, topical analgesics, anesthetics, cryotherapy, and combined therapies, each targeting pain through distinct mechanisms (25). Integrating these approaches may offer enhanced pain relief and promote healing.

In conclusion, the results of this review suggest a great degree of uncertainty on the impact of PRF on pain severity associated with alveolar osteitis. This may be due to the substantial heterogeneity of therapeutic options used in the control group and the variability of surgical techniques and surgical sites used to perform dental extractions. Despite these findings, this review also demonstrates that PRF improves alveolar healing, reduces analgesic use in patients, and likely increases overall clinical efficacy, making it a valuable alternative in the treatment of alveolar osteitis. Although these results showed positive effects, further randomized clinical trials with standardized methodologies must be performed to validate these findings.

## Figures and Tables

**Table 1:** Characteristics of the included trials.

Trial	Control intervention (n)	Follow-up (D)	RPM x Min	Outcomes assessed	Inclusion criteria	Exclusion criteria
Keshini 2020 (8)	Alvogyl	10	3000 rpm for 10 minutes	Pain intensity, Soft tissue healing	Patients diagnosed for dry socket.	Children (<18 y-o) and old people (>60 y-o).
Hussain 2018 (26)	Zinc oxide eugenol (ZOE)	7	3000 rpm for 10 minutes	Pain intensity, Swelling degree, Soft tissue healing	Patients diagnosed with alveolar osteitis, with the surgical site free of active infection.	Systemic diseases or compromised immunity; smokers; pregnant and lactating women.
Yuce 2019 (24)	Saline colution	7	1300 rpm for 8 minutes	Pain intensity, Soft tissue healing, Analgesic usage and Bone density	Adult patients diagnosed with alveolar osteitis and no systemic disease.	Smokers; pregnancy or lactation; dental infection; radiation therapy or chemotherapy.
Wang 2023 (32)	Iodoform gauze	7	1500 rpm for 8 minutes	Clinical efficacy, Pain intensity, Analgesic usage	Patients diagnosed with localized alveolar osteitis and no systemic disease	Patients with systemic or acute/chronic infectious diseases; smokers and alcohol drinkers.

**Table 2:** Characteristics of study participants of the included studies.

Trial	Mean age (years)	Sex (%)	PRF (n)	Control (n)	Extraction site
Keshini 2020 (8)	NR	NR	15	15	NR
Hussain 2018 (26)	35.2	Male (22.5) Female (77.5)	20	20	Mandibular molars
Yuce 2019 (24)	31.2	Male (45) Female (55)	20	20	Mandibular third molar
Wang 2023 (32)	33.5	Male (35) Female (65)	30	30	Mandibular third molar

**Table 3:** Summary of findings table (GRADE SoF table).

PRF for the treatment of alveolar osteitis						
Outcomes	Relative effect (95% CI) —-- Patients/ studies	Absolute effect*			Certainty of evidence (GRADE)	Key messages
		WithoutPRF	WithPRF	Difference (CI 95%)		
Pain severity (day 3)	170 patients/ 4 trials	8.33	6.67	MD 1.66 fewer (4.11 fewer to 0.78 more)	⨁○○○^1,2^ VERY LOW	The evidence is very uncertain about the effect of platelet-rich on pain severity measured on day 3 (very low certainty evidence)
Pain severity (day 7)	140 patients/ 3 trial	8.33	6.76	MD 1.57 lower (4.01 lower to 0.88 higher)	⨁○○○^1,2^ VERY LOW	The evidence is very uncertain about the effect of platelet-rich on pain severity measured on day 7 (very low certainty evidence)
Alveolar socket healing (day 7)	100 patients/ 2 trial	SMD 2.25 higher (1.7 higher to 2.8 higher)			⨁⨁⨁○^2^ MODERATE	The use of platelet-rich fibrin increases alveolar healing.
Analgesic use(day 7)	100 patients/ 2 trial	13.5^4^	5.65	MD 7.85 lower (11.93 lower to 3.77 lower)	⨁⨁⨁○^3^ MODERATE	The use of platelet-rich fibrin reduces analgesic use.
Clinical efficacy(day 7)	RR 1.56 (1.14 to 2.12) 60 patients/ 1 trial	600 per 1,000	936 per 1,000 (684 to 1,000)	336 more per 1,000 (84 more to 672 more)	⨁⨁⨁○^2^ MODERATE	The use of platelet-rich fibrin likely increases clinical efficacy.
Swelling (day 3)	40 patients/ 1 trial	0.4	0.4	MD 0.00 (0.36 lower to 0.36 higher)	⨁⨁○○^3^ LOW	The use of platelet-rich fibrin results in little to no difference in swelling degree measured on day 3 (low certainty evidence)
Patients	Individuals with alveolar osteitis					
Intervention	PRF (as defined by studies)					
Comparison	Standard treatment (as defined by studies)					

Margin of error: 95% confidence interval (CI); MD: Mean difference; SMD: Standard mean difference; GRADE: Grading of Recommendations Assessment, Development and Evaluation. *The risk without PRF is based on the risk in the control group of the trials. The risk with PFR (and its margin of error) is calculated from relative effect (and its margin of error). ^1^ The certainty of the evidence was downgraded two levels for imprecision due to the small number of trial participants and because the boundaries of 95% confidence interval estimated crosses the threshold of the absolute effect (minimal important difference for VAS scale 2-3 points). ^2^ The certainty of the evidence was downgraded one level for inconsistency because a substantial (>60%) or considerable (>75%) heterogeneity was identified in the pooled results (I2 = X%). ^3^ The certainty of the evidence was downgraded one level for imprecision due to the small number of trial participants.^4^ Baseline risk from the study with the highest weighting included in the analysis.
